# The impact of ultrasound-guided erector spinae plane block combined with paravertebral block on postoperative rebound pain following thoracoscopic lobectomy

**DOI:** 10.3389/fmed.2025.1651245

**Published:** 2025-08-07

**Authors:** Yihang He, Dongxu Chen, Youbo Zuo, Jing Lin

**Affiliations:** ^1^Department of Anesthesiology, Affiliated Hospital of North Sichuan Medical College, Nanchong, Sichuan, China; ^2^Department of Anesthesiology, West China Second University Hospital, Sichuan University, Chengdu, Sichuan, China

**Keywords:** rebound pain, paravertebral block, erector spinae plane block, thoracoscopic surgery, postoperative recovery quality

## Abstract

**Background:**

Thoracic paravertebral block (TPVB) is the mainstream analgesic regimen for post-video-assisted thoracoscopic surgery (VATS) pain management. However, rebound pain frequently emerges once the block effect subsides. Given that the erector spinae plane block (ESPB) may modulate the incidence of rebound pain through its mechanism of local anesthetic diffusion into the paravertebral space, this study sought to evaluate whether combining TPVB with ESPB could effectively reduce postoperative rebound pain in VATS patients.

**Methods:**

A total of 110 patients scheduled for elective video-assisted thoracoscopic lobectomy were enrolled and randomly allocated via a random number table to either the TPVB group (Group P, *n* = 55) or the TPVB combined with ESPB group (Group PE, *n* = 55). In Group P, TPVB was performed under oblique axial scanning at the T5 level using the in-plane technique, with 20 mL of 0.5% ropivacaine administered. In Group PE, TPVB was first performed with 10 mL of 0.5% ropivacaine; the needle was then withdrawn and repositioned with its tip deep to the erector spinae muscle at the transverse process level, followed by injection of 10 mL of 0.5% ropivacaine for ESPB. The primary outcome was the incidence of rebound pain within 24 h postoperatively. Secondary outcomes included: Numeric Rating Scale (NRS) scores at rest (quiet supine position) and during activity (coughing and expectoration) on postoperative day 1 morning (D1 am), evening (D1 pm), day 2 morning (D2 am), and evening (D2 pm); time to first rebound pain within 24 h; Modified Rebound Pain Scale (MRPS) score; Quality of Recovery-15 (QoR-15) scores on postoperative days 1 and 2; total sufentanil consumption via patient-controlled intravenous analgesia (PCIA) over 48 h; number of rescue analgesia doses administered in the ward; postoperative hospital stay; patient satisfaction score at discharge; and postoperative complication rate.

**Results:**

Compared with Group P, Group PE had a significantly lower incidence of rebound pain within 24 h postoperatively (23.64% vs. 47.27%, *p* = 0.010) and a significantly reduced MRPS score (3.06 ± 1.75 vs. 3.84 ± 2.05; *p* = 0.035). Additionally, Group PE had lower activity-related NRS scores on D1 am (*p* = 0.010), D1 pm (*p* < 0.001), and D2 pm (*p* = 0.031), as well as a lower resting NRS score on D1 am (*p* = 0.048). Furthermore, Group PE showed higher QoR-15 scores on both postoperative days 1 and 2 (*p* < 0.05), reduced 48-h PCIA sufentanil consumption (*p* = 0.002), fewer rescue analgesia requirements (*p* = 0.048), and a shorter postoperative hospital stay (*p* < 0.001).

**Conclusion:**

Compared with TPVB alone, the combination of TPVB and ESPB significantly reduces the incidence of postoperative rebound pain, prolongs analgesic duration, and improves the quality of postoperative recovery.

## Introduction

Video-assisted thoracoscopic surgery (VATS) has emerged as the preferred alternative to open thoracotomy, owing to its significant advantages in reducing surgical trauma and inflammatory responses, as well as shortening hospital stays (1, 2). However, postoperative pain remains a critical issue affecting patient recovery after VATS. Studies have indicated that postoperative pain primarily stems from chest wall incisions, intercostal nerve injuries, stimulation by chest drain tubes, and pleural inflammation. Its intensity reaches moderate-to-severe levels, with a particular peak within the first 24 h postoperatively (3). This pain not only impairs patients’ ability to cough and expectorate—thereby increasing the risk of pulmonary infection and atelectasis—but may also trigger chronic neuropathic pain, severely compromising postoperative quality of life (4).

In VATS, regional nerve blocks constitute an essential component of combined anesthesia and postoperative multimodal analgesia. Thoracic paravertebral block (TPVB), which precisely blocks nociceptive transmission through the thoracic spinal nerves and offers advantages such as reliable analgesic efficacy, has become a mainstream choice for post-thoracoscopic analgesia (5). In recent years, the erector spinae plane block (ESPB) has attracted attention due to its simplicity and high safety profile, though its analgesic effectiveness remains a subject of debate (6).

Nevertheless, clinical observations and studies have revealed that some patients experience a sudden intensification of pain after the nerve block effect wears off (7, 8), a phenomenon termed “rebound pain.” Rebound pain is defined as transient, acute, severe postoperative pain that occurs after the regression of a peripheral nerve block. Specifically, it refers to a sudden transition from well-controlled pain (Numerical Rating Scale, NRS ≤ 3) to severe pain (NRS ≥ 7) in patients receiving regional anesthesia, presenting as intense burning pain at rest or during movement. This pain typically lasts 2–6 h and occurs more frequently at night (9). It has been primarily reported in orthopedic surgeries (e.g., shoulder, ankle procedures) involving upper limb brachial plexus blocks or lower limb sciatic/femoral nerve blocks.

Although rebound pain is most commonly observed in orthopedic surgery, recent studies have indicated that it also occurs after TPVB in VATS, with an incidence as high as 33.3% (10). This may be associated with the injection of local anesthetics (LA) into the paravertebral space (PVS), which contains intercostal spinal nerves, dorsal root ganglia, and the sympathetic chain (11–13). While single-injection TPVB can provide adequate analgesia, it often results in high concentrations of LA localized at a single vertebral level, potentially leading to rapid drug dissipation and triggering postoperative rebound pain. ESPB exerts its effects by facilitating the diffusion of LA into the paravertebral space (14, 15). This indirect mechanism of action may delay its onset but could potentially reduce the incidence of postoperative rebound pain.

Previous studies have demonstrated that combining TPVB with ESPB can shorten the onset time of the block, prolong the duration of nerve blockade, reduce the incidence of intraoperative hypotension during VATS, and decrease the risk of chronic postoperative pain (16, 17). However, the impact of this combined block on the incidence of postoperative rebound pain in patients has not been investigated. In this study, we aim to explore whether the combination of TPVB and ESPB can effectively reduce the incidence of postoperative rebound pain in patients undergoing VATS.

## Materials and methods

### Study design and patient enrollment

This study was approved by the Ethics Committee of the Affiliated Hospital of North Sichuan Medical College (Approval No: 2024ER195-1) and registered with the Chinese Clinical Trial Registry (ChiCTR2400084759). The clinical trial followed the Declaration of Helsinki, the Good Clinical Practice (GCP) for Drug Clinical Trials issued by the State Drug Administration (SDA) and other relevant regulations. Written informed consent was obtained from all participating patients.

Patients scheduled for thoracoscopic lobectomy between May 2024 and December 2024 were recruited. The inclusion criteria were as follows: age 18 to 65 years, ASA classification I to III, and scheduled for elective unilateral VATS. Exclusion criteria included: (1) body mass index greater than 30 kg/m^2^; (2) coagulation disorders, (e.g., platelet count below normal or prolonged clotting time); (3) history of opioid misuse, defined as persistent or recurrent use beyond medical guidelines and for non-medical purposes; (4) prior diagnosis of chronic pain; (5) allergies to local anesthetics or analgesics; (6) infection at the injection site; (7) inability to comprehend or respond to pertinent questions.

### Randomization and blinding

Prior to initiating the study, an independent research assistant utilized a computer-generated random number table to assign participants into two equal groups. This allocation process was masked by using sequentially labeled opaque sealed envelopes. Before anesthesia administration, another assistant, who was not part of the study, opened one of these envelopes to reveal the group assignment. The attending anesthesiologist then performed the designated nerve block anesthesia (either TPVB or ESPB combined with TPVB) as per the group allocation. Throughout the data collection period, personnel involved in postoperative data collection (pain scores NRS, MRPS, QoR-15, satisfaction), administration of rescue analgesia, and assessment of complications in the PACU and surgical ward were blinded to group allocation. Patients were informed that they were receiving one of two effective regional analgesic techniques being compared, without specifying the details of the blocks, to minimize expectation bias regarding pain relief or rebound pain.

### Intervention

Patients were randomly assigned to one of two groups: the TPVB group (Group P) or the TPVB combined with ESPB group (Group PE). Nerve blocks were administered following anesthesia induction in both groups. All blocks were performed by a certified anesthesiologist experienced in ultrasound using US guidance (Mindray M9 Ultrasound System) and a high-frequency linear ultrasound probe. All patients were positioned in a lateral decubitus manner, and the needle was inserted at the level of the T5 transverse process.

**Figure 1:**
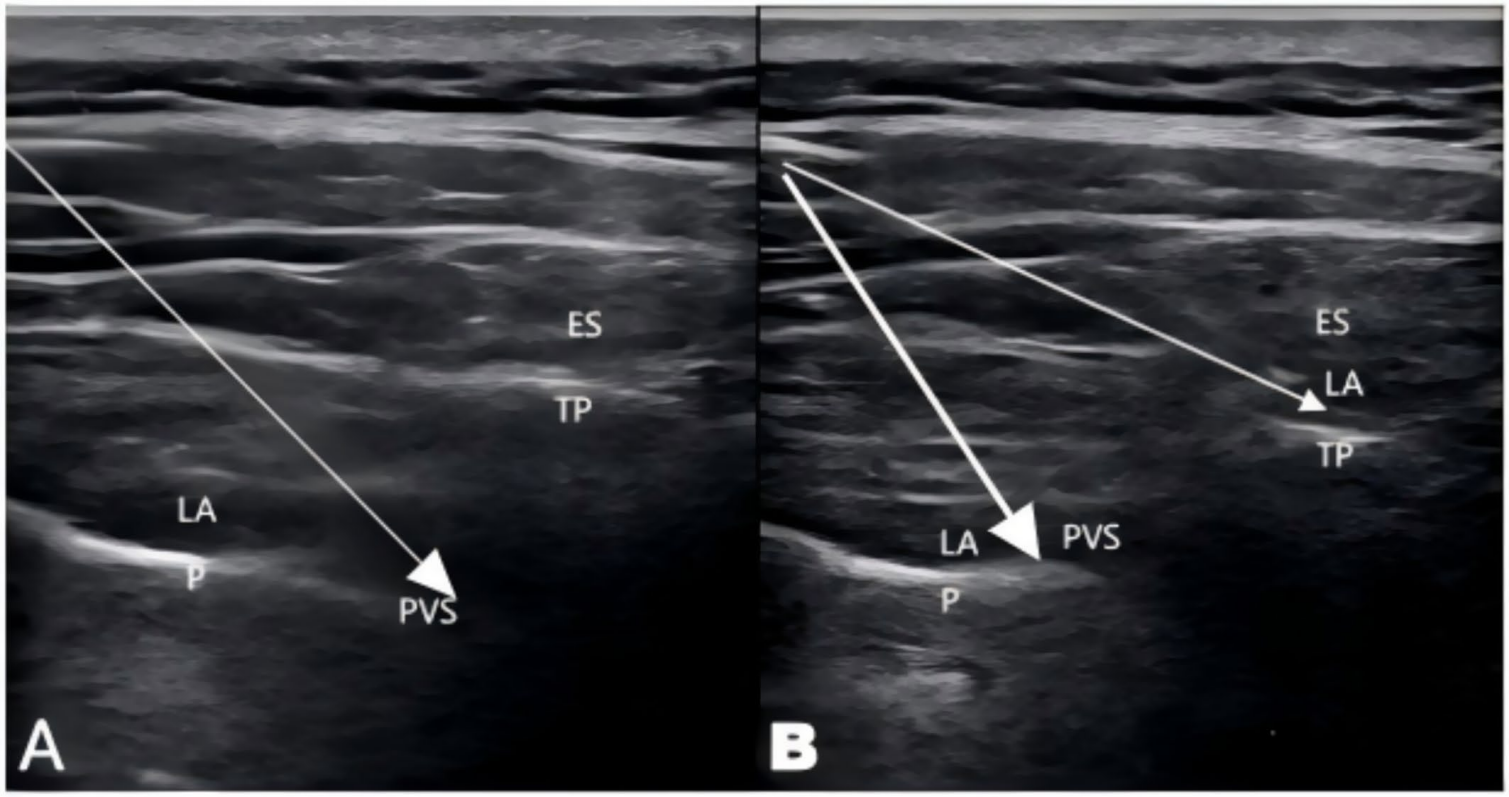
Procedures for the TPVB and the TPVB+ESPB. **(A)** The ultrasound imaging after injection of TPVB and pleura depression was observed. **(B)** The ultrasound imaging of TPVB+ESPB after injection at erector spinae muscles. P, Pleura; PVS, Paravertebral space; TP, Transverse process; ES, Erector spinae; LA, Local anesthetics; White arrows indicate the needle injection path.

For TPVB, as previously described (18), 20 mL of 0.5% ropivacaine was administered slowly. Successful blockade was indicated by the displacement of the pleura (Figure 1A). In the group PE, TPVB combined with ESPB was performed through a single puncture. A high-frequency linear array transducer was used to locate the T5 paravertebral space, the tip of the T5 transverse process, overlying the erector spinae muscle, apex of the TPVS, and pleura were identified. A 22-gauge block needle was inserted using an in-plane technique. After confirming no blood return upon aspiration, 10 mL of 0.5% ropivacaine was slowly injected, with successful blockade indicated by the pleura being displaced deeper by the local anesthetic. The needle was then withdrawn and repositioned at the surface of the T5 transverse process. An additional 10 mL of 0.5% ropivacaine was injected above the T5 transverse process, with local anesthetic dispersion visualized under ultrasound (Figure 1B).

### Anesthesia management

All patients were required to undergo routine fasting and abstinence from water. Upon entering the operating room, the patient’s basic information was verified, and monitoring devices were connected to measure electrocardiogram, oxygen saturation, and non-invasive blood pressure. Peripheral intravenous access was established, and anesthesia was standardized for both groups of patients. General anesthesia was induced using propofol (2–3 mg/kg), sufentanil 0.5 μg/kg, and cisatracurium 0.2 mg/kg, followed by the insertion of a double-lumen tube. Mechanical ventilation was initiated with a constant flow volume-controlled mode, setting tidal volume at 6–8 mL/kg, and adjusting ventilation frequency to maintain end-tidal carbon dioxide pressure between 35 and 45 mmHg, with airway pressure kept below 30 cm H₂O. Anesthesia was maintained with 1–2% sevoflurane inhaled in 50% oxygen, propofol infusion at 2–6 mg/(kg.h), to maintain bispectral index (BIS) values between 40 and 60. Intermittent intravenous injections of sufentanil (based on blood pressure and heart rate) and cisatracurium (0.05 mg/kg every 30 min) were also administered. Intraoperative hypotension (defined as systolic pressure < 90 mmHg or a decrease of more than 20% from baseline) was managed with an intravenous injection of ephedrine 6 mg. If the heart rate (HR) dropped below 50 beats per minute, atropine 0.5 mg was administered intravenously. The intraoperative fluctuations in heart rate and blood pressure were maintained within 20% of the baseline values. Upon meeting the extubation criteria at the conclusion of the surgery, the double-lumen endotracheal tube was removed, and the patient was transferred to the Post-Anesthesia Care Unit (PACU) for observation.

Postoperatively, both groups of patients received patient-controlled intravenous analgesia (PCIA) pumps, with a formulation consisting of sufentanil 150 μg, butorphanol 8 mg, and tropisetron 5 mg, diluted in 150 mL of normal saline. The initial dose was set to zero, the continuous infusion rate was 1.5 mL/h, the bolus dose was 3 mL, with a lockout interval of 15 min, and a maximum dosage of 10 mL/h. In the Post-Anesthesia Care Unit (PACU), if a patient reports a pain score of NRS ≥ 4, the anesthesia nurse will administer a bolus dose by activating the button on the patient-controlled intravenous analgesia (PCIA) pump. Following transfer to the surgical ward, should the patient report an NRS score ≥ 4, the rescue analgesia protocol is initiated with the intravenous administration of 100 mg tramadol. Additionally, if moderate to severe postoperative nausea or vomiting (PONV) occurs, 2.5 mg tropisetron will be administered intravenously.

### Outcomes

The primary outcome was the incidence of rebound pain within the first 24 h postoperatively. Rebound pain was monitored hourly by trained ward nursing staff who were blinded to group allocation. Patients were specifically asked about their pain intensity at rest using the NRS. A rebound pain episode was recorded if the patient reported an NRS score ≥ 7, representing a sudden increase from their lowest recorded NRS score (≤ 3) documented upon leaving the PACU.

### Secondary outcomes

The secondary outcome measures included the time to first rebound pain within 24 h postoperatively; the Modified Rebound Pain Score (MRPS), calculated as MRPS = HNRS - LoNRS(PACU), where HNRS represents the highest NRS pain score recorded within 24 h after the regional nerve block, and LoNRS(PACU) represents the lowest NRS pain score recorded within the PACU (19); and NRS pain scores assessed at rest (lying quietly) and during activity (coughing and expectoration) at the following time points: preoperative day 1, upon leaving the PACU, morning (D1am) and evening (D1pm) of postoperative day 1, and morning (D2am) and evening (D2pm) of postoperative day 2. Additional outcomes encompassed the total PCIA sufentanil consumption within 48 h postoperatively; the number of rescue analgesic administrations required on the surgical ward; postoperative hospital length of stay; Quality of Recovery-15 (QoR-15) scores on postoperative days 1 and 2 (20); and patient satisfaction at discharge, rated on a scale where 1 = “Dissatisfied,” 2 = “Somewhat satisfied,” and 3 = “Satisfied” (19); Postoperative complications rate, such as block site hematoma, nausea, vomiting, respiratory depression or hypotension.

### Statistical analysis

The sample size was calculated based on a preliminary trial, which the incidence of rebound pain within 24 h postoperatively was 35% for group P, 10% for group PE. Sample size calculation was performed using PASS software (v.15.0, NCSS, USA), and statistical analysis was conducted utilizing the Chi-square test, with a power of 0.90, and *α* error level of 0.05, along with a 20% follow-up loss consideration, the required sample size was determined to be 55 participants per group.

Data management and statistical analysis were conducted using SAS 9.4 (SAS Institute, Inc., Cary, NC, USA). Qualitative data were expressed as frequencies and percentages (%), and inter-group comparisons performed using the Chi-square or Fisher’s exact test. Normality was evaluated via the Shapiro-Wilk test. For normally distributed data, means and standard deviations (x̄± s) were utilized, and comparisons between groups were made using independent samples t-test. The Cox proportional hazards model was applied for survival analysis of rebound pain across groups. Kaplan–Meier curves for rebound pain were plotted. Non-normally distributed data were presented as median (M) and interquartile range (IQR), with comparisons made using the Mann–Whitney U-test. A difference of *p* ≤ 0.05 was considered statistically significant.

## Results

### Baseline data

**Figure 2:**
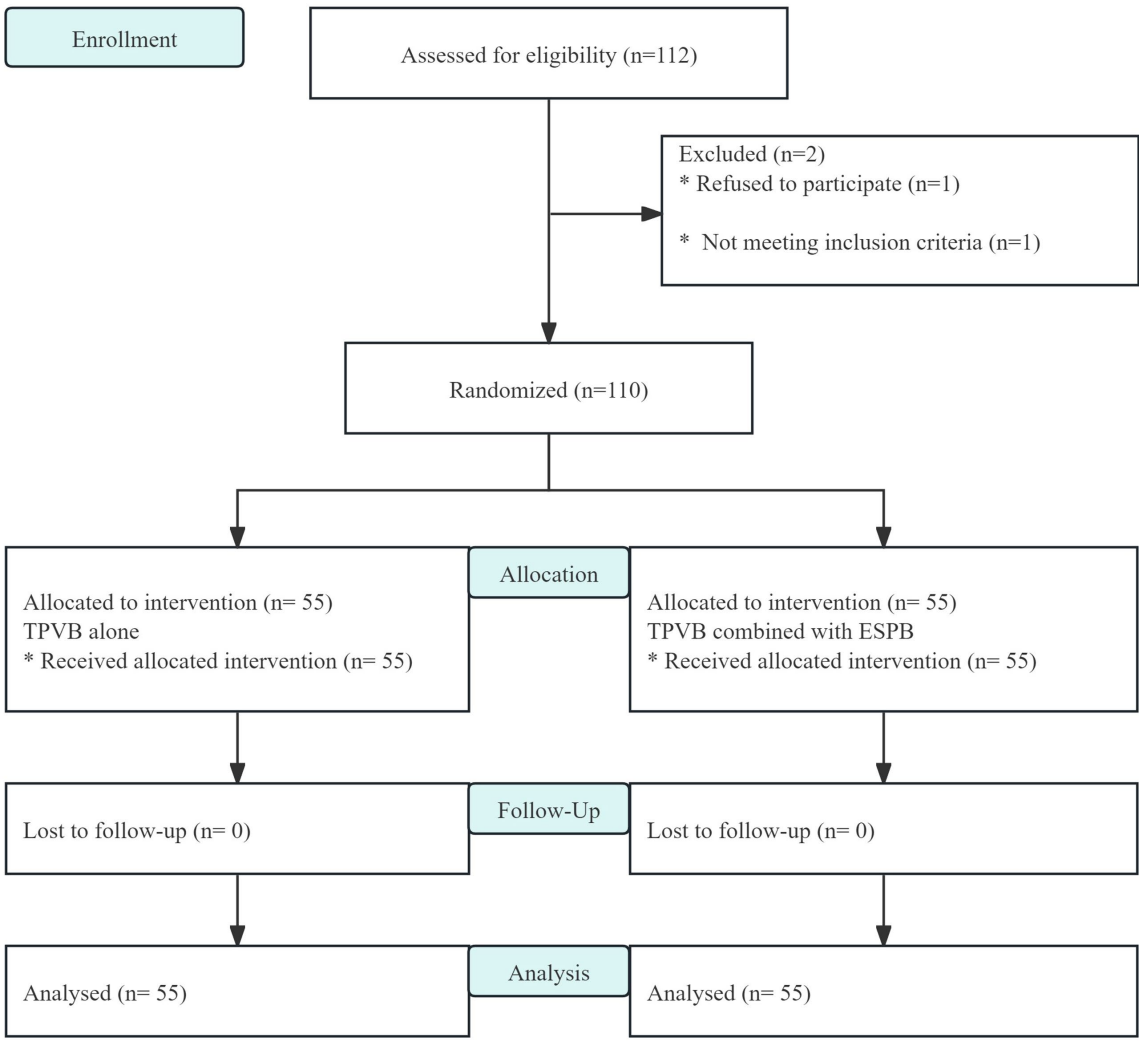
CONSORT flow diagram.

A total of 112 patients were recruited and evaluated in this study, of whom 2 were excluded. The remaining 110 patients were randomly assigned to group P and group PE (n = 55) and entered into the intentional analysis (Figure 2). There were no statistically significant differences in the baseline data between the two groups (Table 1).

**Table 1:** Demographic data and clinical variables.

Variables	Group P (*n* = 55)	Group PE (*n* = 55)	*p*
Gender, *n* (%)			0.055
Male	29 (52.73)	19 (34.55)	
Female	26 (47.27)	36 (65.45)	
Age (year)	56.0 ± 8.8	54.9 ± 11.1	0.568
BMI (kg/m^2^)	24.2 ± 2.9	23.6 ± 3.5	0.344
ASA classification			0.340
II	42 (76.36)	46 (83.64)	
III	13 (23.64)	9 (16.36)	
Surgical site			0.128
Right upper lobe of the lung	19 (34.55)	27 (49.09)	
Right lower lobe of the lung	12 (21.82)	7 (12.73)	
Right middle lobe of the lung	4 (7.27)	3 (5.45)	
Left upper lobe of the lung	9 (16.36)	14 (25.45)	
Left lower lobe of the lung	11 (20.00)	4 (7.27)	
Duration of surgery (min)	136.7 ± 25.4	128.5 ± 21.5	0.071
Intraoperative Sufentanil Dosage(μg)	59.6 ± 4.2	58.1 ± 4,1	0.488
Extubation time (min)	27.3 ± 4.1	27.4 ± 4.1	0.889
Length of stay in PACU (min)	60.2 ± 6.1	61.6 ± 6.5	0.239

Data are expressed as mean ± SDs or number of patients (%) as appropriated.

### Primary outcome

**Figure 3:**
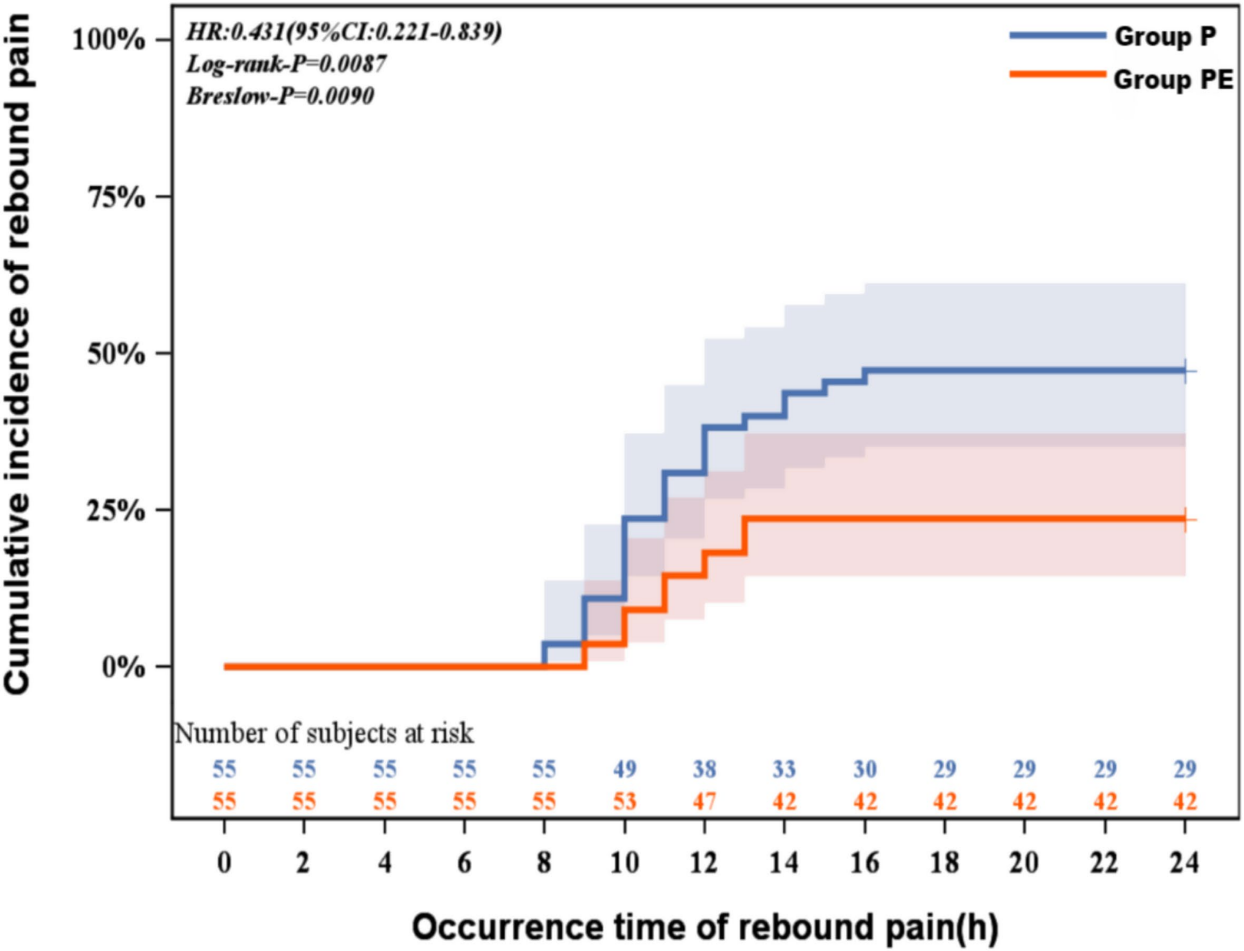
Kaplan-Meier curve for rebound pain survival analysis.

The Kaplan-Meier analysis revealed a significantly lower cumulative incidence of rebound pain in the group PE compared to group P(log-rank *p* = 0.0087; Breslow *p* = 0.0090). The hazard ratio of 0.43 (95% CI: 0.22–0.84) indicates a 57% reduction in rebound pain risk with the combined block. This protective effect emerged within 8 h postoperatively and progressively widened over the observation period, with the most pronounced separation occurring between 8 and 14 h when the TPVB-alone group demonstrated accelerated rebound pain onset (Figure 3). Following adjustment for confounding variables, the group PE demonstrates a 60% reduction in risk compared to group P, with a hazard ratio of 0.41 (95% CI 0.20 to 0.80; *p* = 0.009; Table 2).

**Table 2:** Survival analysis of rebound pain.

Variables	Unadjusted		Adjusted	
	HR (95%CI)	*p*	HR (95%CI)	*p*
Group P	1.00 (ref)		1.00 (ref)	
Group PE	0.43 (0.22 to 0.83)^*^	0.013	0.40 (0.20 to 0.80)^*^	0.009

Adjusted for age, gender and BMI. * indicates statistically significant difference (*p* < 0.05).

### Secondary outcome

**Figure 4:**
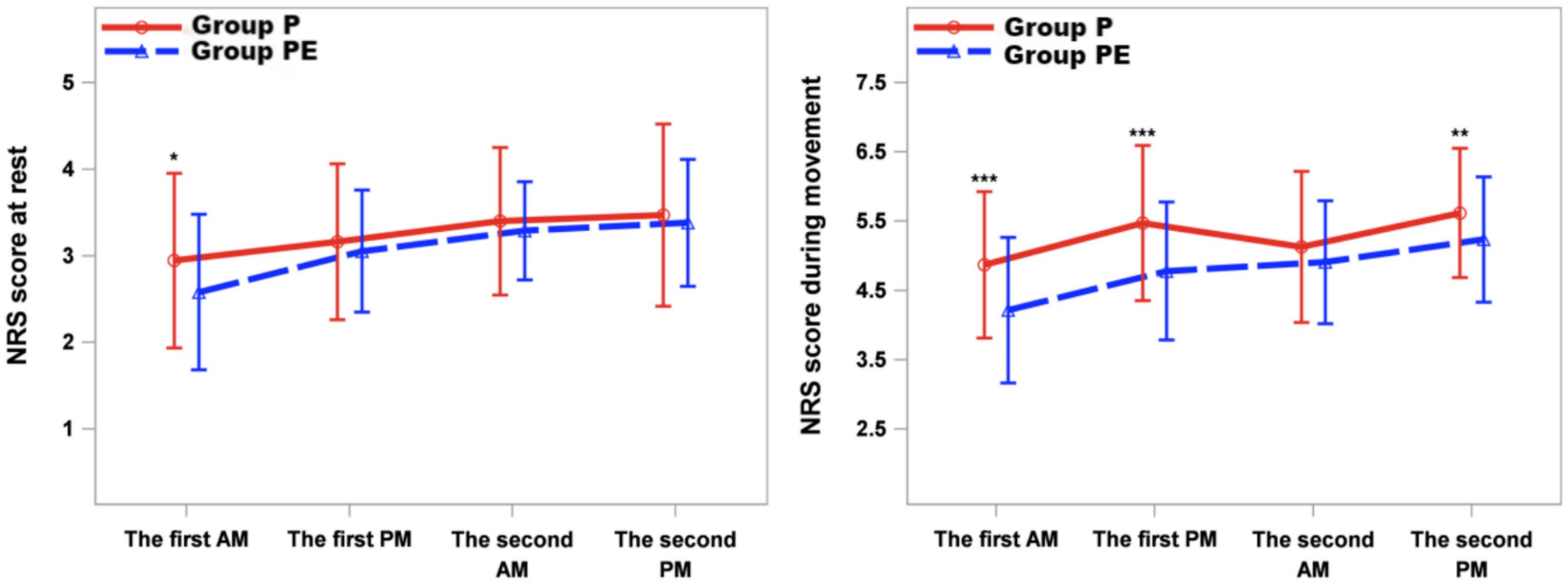
Mean numeric rating scale (NRS) score at rest and while coughing during the first 2 d after surgery. The error bars represent standard error. ^⁎^The difference was significant at 0.05 level.

As shown in Figure 4, NRS pain scores at rest (quiet rest) were significantly lower in the group PE compared to the group P at the D1am time point (*p* = 0.048). Resting NRS scores at other time points within the first 48 h postoperatively did not differ significantly between the two groups. For NRS pain scores during activity (coughing and expectoration), the group PE demonstrated significantly lower scores than the group P at the D1am, D1pm, and D2pm time points (*p* < 0.05) (Figure 4).

Significant differences were observed between the two groups in QoR-15 scores on postoperative days 1 and 2, postoperative hospital length of stay, PCIA sufentanil consumption within 48 h, and the MRPS. Specifically, the group PE demonstrated a significantly higher QoR-15 score than the group P on postoperative day 1 (100.3 ± 10.1 vs. 94.4 ± 7.3, *p* < 0.001). Although statistically significant (*p =* 0.048), the observed difference in rescue analgesia should be interpreted conservatively. The borderline *p*-value and identical median values (0 vs. 1) indicate fragile evidence for clinical meaningfulness. However, no significant differences were observed between group P and PE regarding the time to first rebound pain or patient satisfaction (Table 3).

**Table 3:** Postoperative data comparisons between two groups.

Variables	Group P (*n* = 55)	Group PE (*n* = 55)	*p*
QoR-15 score on postoperative day 1	94.4 ± 7.3	100.3 ± 10.1	<0.001
QoR-15 score on postoperative day 2	99.0 ± 8.8	108.5 ± 11.1	<0.001
Modified Rebound Pain Score (MRPS)	3.84 ± 2.05	3.06 ± 1.75	0.035
Time to First Rebound Pain (h)	11.00 ± 2.04	11.08 ± 1.38	>0.8
PCIA Sufentanil Consumption (μg)	81 (72, 84)	78 (72, 81)	0.002
Rescue Analgesia (ward), *n*	1 (0, 2)	0 (0, 2)	0.048
Postoperative Hospital Stay (days)	5.00 (4.00, 5.00)	3.00 (3.00, 5.00)	<0.001
Patient Satisfaction, *n* (%)			0.368
Dissatisfied (1)	7 (12.73)	6 (10.91)	
Somewhat satisfied (2)	33 (60.00)	27 (49.09)	
Satisfied (3)	15 (27.27)	22 (40.00)	

Data are represented as mean ± SDs, M (IQR) or patients (%) as appropriated.

Postoperative complications are summarized in Table 4. No significant intergroup differences were observed in the incidence of nausea/vomiting, postoperative hypotension, hematoma at block site, and respiratory depression after surgery.

**Table 4:** Postoperative complications.

Variables	Group P (*n* = 55)	Group PE (*n* = 55)	*p*
Nausea/vomiting, *n* (%)	8 (14.50)	5 (9.10)	0.38
Hypotension, *n* (%)	3 (5.50)	2 (3.60)	0.65
Hematoma at block site, *n* (%)	0 (0.00)	0 (0.00)	–
Respiratory depression, *n* (%)	0 (0.00)	0 (0.00)	–

Data are represented as patients (%) as appropriated.

## Discussion

This single-center randomized controlled trial demonstrates that, compared to TPVB alone, the combination of TPVB and ESPB significantly reduced the incidence of rebound pain from 47.3 to 23.6%, without compromising intraoperative and postoperative analgesic efficacy. Additionally, the combined block improved the QoR-15 scores on postoperative days 1 and 2.

Acute pain following thoracic surgery is extremely severe and may progress to chronic pain. It has been reported that the incidence of chronic pain at 3 and 6 months post-thoracotomy is 57 and 47%, respectively, ranking among the highest of all surgical procedures (21, 22). Despite the introduction of VATS, nearly 16% of patients still experience moderate-to-severe acute pain within 48 h postoperatively (23). The optimal postoperative pain management strategy for VATS remains uncertain. Existing systematic reviews and guidelines for post-VATS pain management recommend regional analgesic techniques such as paravertebral block, serratus anterior plane block, and erector spinae plane block (24, 25). However, even as the analgesic effect of nerve blocks wanes, there remains a 30–45% chance of severe rebound pain occurring (26–28). Notably, the incidence of rebound pain in the TPVB group of this study (47.3%) differed significantly from the 33.3% incidence reported by Zeng et al. (10) for TPVB-associated rebound pain after VATS. This clinical discrepancy may be related to differences in the pharmacological parameters of the local anesthetics used. Specifically, our protocol employed 20 mL of 0.5% ropivacaine for the blocks, presenting a gradient difference in both concentration and volume compared to the 15 mL of 0.25% ropivacaine used in the study by Zeng et al.

Rebound pain, recognized as a side effect of regional anesthesia (RA), manifests as intensified pain perception following the regression of local anesthetic effects. This pain is often described as excruciating and intractable, exerting profound negative effects on mental health, recovery quality, and activities of daily living. Although the phenomenon has garnered research attention over the past decade, its precise mechanisms remain unelucidated, and specific treatment guidelines have not been established (8, 29). RA suppresses the amplification of neuronal activity and responsiveness in the spinal dorsal horn, a process known as central sensitization (30). Conversely, peripheral nerve blocks (PNBs) have limited impact on peripheral sensitization, and inflammatory processes persist unabated in the absence of systemic pharmacologic intervention (31). Consequently, when the effect of a PNB regresses, nociceptive signals originating from the hyperalgesic zone at the injury site manifest as rebound pain. Furthermore, local anesthetics themselves may possess neurotoxic and cytotoxic properties (32–34). The combination of ESPB and TPVB employed in this study may reduce the exposure of nerve roots to local anesthetics and delay their rapid dissipation, thereby lowering the incidence of rebound pain.

This study found that TPVB combined with ESPB significantly reduced the incidence of postoperative rebound pain compared to TPVB alone. Other methods to reduce rebound pain include continuous PNB catheter techniques, adjunctive medications for single-shot PNBs, and multimodal analgesia regimens (9, 29, 35). However, continuous regional blocks are associated with issues such as catheter dislodgement, infection, patient discomfort, and increased management time (36, 37). The TPVB+ESPB combination offers a novel option for rebound pain prevention.

The study by Fu et al. (38) showed that the combined application of TPVB and ESPB after VATS reduced hydromorphone consumption within 12 and 48 h compared to a single block. In non-intubated VATS, the combined block also optimizes surgical conditions by suppressing the cough reflex (39). Consistent with these findings, our study observed reductions in PCIA sufentanil consumption within 48 h, the number of rescue analgesic administrations on the ward, and NRS scores during coughing within 48 h in the group PE compared to the group P.

The study data revealed a significant difference in MRPS between the PE and P groups (3.06 ± 1.75 vs. 3.84 ± 2.05, *p* = 0.035). Previous research by Barry et al. (9) confirmed that increased MRPS scores directly correlate with decreased patient satisfaction with postoperative analgesia. The lower MRPS levels in the group PE suggest less severe rebound pain. Notably, despite the higher incidence of rebound pain in the group P, 87.27% of these patients still rated their overall pain management as “satisfied” or “somewhat satisfied,” a result not significantly different from the group PE and consistent with prior research conclusions (9). This apparent discrepancy may arise from several factors. Firstly, while rebound pain is distressing, the overall analgesic protocol (including PCIA and rescue tramadol) was effective in managing pain for most patients in both groups after the initial rebound episode, leading to comparable final satisfaction. Secondly, satisfaction is a multidimensional construct influenced by factors beyond pain control alone, such as communication with staff, overall hospital experience, and achievement of recovery milestones (19). Thirdly, the 3-point satisfaction scale used, while practical, may lack the sensitivity to detect subtle differences in satisfaction levels compared to more granular scales like a Visual Analogue Scale (VAS) for satisfaction. Future studies could benefit from using more sensitive satisfaction metrics.

This study utilized the QoR-15 scale to assess the quality of recovery after anesthesia and surgery (20). According to the study by Et T et al. (40), the reduction in rebound pain incidence leads to significantly less moderate-to-severe pain, providing patients with better control and a more comfortable experience. Our finding of significantly better QoR-15 scores in the group PE on postoperative days 1 and 2 indicates that the combined block effectively enhances postoperative recovery quality and promotes early rehabilitation, thereby shortening hospital length of stay. The shorter hospital stay in the group PE likely reflects synergistic benefits of improved analgesia enabling earlier mobilization, reduced opioid-related side effects, and enhanced overall recovery—though surgical factors and complication rates also play critical roles. We also posit that rebound pain has a particularly disruptive effect on early postoperative sleep quality and quality of life.

This study has several limitations: First, the nerve blocks were performed after anesthesia induction, and the dermatomal level of TPVB and ESPB analgesia was not routinely verified, although ultrasound was used to confirm local anesthetic spread. Concurrent dermatomal sensory examinations were not performed postoperatively. Future studies should correlate sensory loss duration with rebound pain onset. Second, to avoid interference from other factors, additional specific rebound pain prevention measures were not extensively employed. In clinical practice, adjuncts like dexamethasone added to local anesthetics or postoperative multimodal analgesia incorporating agents such as oxycodone could be used to minimize rebound pain incidence. Finally, this study focused only on short-term rebound pain following the nerve blocks and did not observe long-term chronic postoperative pain, further investigations with long-term follow-up are warranted to validate the persistence of the outcomes.

## Conclusion

In conclusion, for patients undergoing thoracoscopic lobectomy, the combination of ESPB and TPVB provides superior postoperative pain relief, reduces the incidence of rebound pain, shortens hospital stays, and improves the quality of recovery compared to TPVB alone.

## Data Availability

The raw data supporting the conclusions of this article will be made available by the authors, without undue reservation.

## References

[1] Bendixen M Jørgensen OD Kronborg C Andersen C Licht PB. Postoperative pain and quality of life after lobectomy via video-assisted Thoracoscopic surgery or anterolateral thoracotomy for early stage lung Cancer: a randomised controlled trial. Lancet Oncol. (2016) 17:836–44. doi: 10.1016/s1470-2045(16)00173-x, PMID: 27160473

[2] Leshnower BG Miller DL Fernandez FG Pickens A Force SD. Video-assisted Thoracoscopic surgery Segmentectomy: a safe and effective procedure. Ann Thorac Surg. (2010) 89:1571–6. doi: 10.1016/j.athoracsur.2010.01.061, PMID: 20417779

[3] Zhang Y Zhou R Hou B Tang S Hao J Gu X. Incidence and risk factors for chronic postsurgical pain following video-assisted Thoracoscopic surgery: a retrospective study. BMC Surg. (2022) 22:76. doi: 10.1186/s12893-022-01522-1, PMID: 35236334 PMC8892711

[4] Wildgaard K Ringsted TK Hansen HJ Petersen RH Kehlet H. Persistent postsurgical pain after video-assisted thoracic surgery--an observational study. Acta Anaesthesiol Scand. (2016) 60:650–8. doi: 10.1111/aas.12681, PMID: 26792257

[5] Jiang T Mo X Zhan R Zhang Y Yu Y. Regional block techniques for pain management after video-assisted Thoracoscopic surgery: a covariate-adjusted Bayesian network Meta-analysis. Wideochir Inne Tech Maloinwazyjne. (2023) 18:52–68. doi: 10.5114/wiitm.2023.124407, PMID: 37064553 PMC10091915

[6] Pang J You J Chen Y Song C. Comparison of erector spinae plane block with paravertebral block for Thoracoscopic surgery: a Meta-analysis of randomized controlled trials. J Cardiothorac Surg. (2023) 18:300. doi: 10.1186/s13019-023-02343-w, PMID: 37891645 PMC10612156

[7] Korkusuz M Basaran B Et T Bilge A Yarimoglu R Kurucay Y. The effects of dexamethasone added to Ilioinguinal/Iliohypogastric nerve (Iin/Ihn) block on rebound pain in inguinal hernia surgery: a randomized controlled trial. Hernia. (2023) 27:1571–80. doi: 10.1007/s10029-023-02841-9, PMID: 37477788

[8] Streb T Schneider A Wiesmann T Riecke J Schubert AK Dinges HC. Rebound pain-from definition to treatment. Anaesthesiologie. (2022) 71:638–45. doi: 10.1007/s00101-022-01120-z, PMID: 35513729 PMC9352600

[9] Barry GS Bailey JG Sardinha J Brousseau P Uppal V. Factors associated with rebound pain after peripheral nerve block for ambulatory surgery. Br J Anaesth. (2021) 126:862–71. doi: 10.1016/j.bja.2020.10.035, PMID: 33390261

[10] Zeng X Zhang X Jiang W Zhou X. Efficacy of intravenous administration of Esketamine in preventing and treating rebound pain after thoracic paravertebral nerve block: a prospective randomized, double-blind, placebo-controlled trial. Drug Des Devel Ther. (2024) 18:463–73. doi: 10.2147/dddt.S448336, PMID: 38384750 PMC10880457

[11] Ruscio L Renard R Lebacle C Zetlaoui P Benhamou D Bessede T. Thoracic paravertebral block: comparison of different approaches and techniques. A study on 27 human cadavers. Anaesth Crit Care Pain Med. (2020) 39:53–8. doi: 10.1016/j.accpm.2019.04.003, PMID: 30978401

[12] Zeng W Zhang J Huang L Tang Z. Analgesic effect of thoracic paravertebral block on patients undergoing Thoracoscopic lobectomy under general anesthesia. Pak J Med Sci. (2023) 39:1774–8. doi: 10.12669/pjms.39.6.7937, PMID: 37936771 PMC10626110

[13] Zhang S Liu Y Liu X Liu T Li P Mei W. Infrared thermography for assessment of thoracic paravertebral block: a prospective observational study. BMC Anesthesiol. (2021) 21:168. doi: 10.1186/s12871-021-01389-4, PMID: 34116642 PMC8194215

[14] Choi YJ Kwon HJ O J Cho TH Won JY Yang HM. Influence of injectate volume on paravertebral spread in erector spinae plane block: an endoscopic and anatomical evaluation. PLoS One. (2019) 14:e0224487. doi: 10.1371/journal.pone.0224487, PMID: 31658293 PMC6816541

[15] Coviello A Vargas M Castellano G Maresca A Servillo G. Ultrasound-guided erector spinae plane block (us-Espb)-anesthetic block: case report. Clin Case Rep. (2020) 8:2885–8. doi: 10.1002/ccr3.3253, PMID: 33363844 PMC7752551

[16] Zengin M Alagöz A Sazak H Ülger G Baldemir R Şentürk M. Comparison of efficacy of erector spinae plane block, thoracic paravertebral block, and erector spinae plane block and thoracic paravertebral block combination for acute pain after video-assisted Thoracoscopic surgery: a randomized controlled study. Minerva Anestesiol. (2023) 89:138–48. doi: 10.23736/s0375-9393.22.16639-3, PMID: 35766959

[17] Zhang L Hu Y Liu H Qi X Chen H Cao W. Analgesic efficacy of combined thoracic paravertebral block and erector spinae plane block for video-assisted thoracic surgery: a prospective randomized clinical trial. Med Sci Monit. (2023) 29:e940247. doi: 10.12659/msm.940247, PMID: 37408302 PMC10334846

[18] Shibata Y Nishiwaki K. Ultrasound-guided intercostal approach to thoracic paravertebral block. Anesth Analg. (2009) 109:996–7. doi: 10.1213/ane.0b013e3181af7e7b 19690285

[19] Zhu Y Li Q Liu G Sheng F Zhang X Jiang L. Effects of esketamine on postoperative rebound pain in patients undergoing unilateral total knee arthroplasty: a single-center, randomized, double-blind, placebo-controlled trial protocol. Front Neurol. (2023) 14:1179673. doi: 10.3389/fneur.2023.1179673, PMID: 37181565 PMC10174246

[20] Liu Q Lin JY Zhang YF Zhu N Wang GQ Wang S. Effects of epidural combined with general anesthesia versus general anesthesia on quality of recovery of elderly patients undergoing laparoscopic radical resection of colorectal Cancer: a prospective randomized trial. J Clin Anesth. (2020) 62:109742. doi: 10.1016/j.jclinane.2020.109742, PMID: 32088534

[21] Capuano P Hileman BA Martucci G Raffa GM Toscano A Burgio G. Erector spinae plane block versus paravertebral block for postoperative pain management in thoracic surgery: a systematic review and meta-analysis. Minerva Anestesiol. (2023) 89:1042–50. doi: 10.23736/S0375-9393.23.17510-9, PMID: 37671541

[22] Clephas PRD Hoeks SE Singh PM Guay CS Trivella M Klimek M. Prognostic factors for chronic post-surgical pain after lung and pleural surgery: a systematic review with meta-analysis, meta-regression and trial sequential analysis. Anaesthesia. (2023) 78:1005–19. doi: 10.1111/anae.16009, PMID: 37094792

[23] Sun K Liu D Chen J Yu S Bai Y Chen C. Moderate-severe postoperative pain in patients undergoing video-assisted Thoracoscopic surgery: a retrospective study. Sci Rep. (2020) 10:795. doi: 10.1038/s41598-020-57620-8, PMID: 31964955 PMC6972772

[24] Feray S Lubach J Joshi GP Bonnet F Van de Velde M. Prospect guidelines for video-assisted thoracoscopic surgery: a systematic review and procedure-specific postoperative pain management recommendations. Anaesthesia. (2022) 77:311–25. doi: 10.1111/anae.15609, PMID: 34739134 PMC9297998

[25] Spaans LN Bousema JE Meijer P Bouwman RAA van den Broek R Mourisse J. Acute pain management after thoracoscopic lung resection: a systematic review and explorative meta-analysis. Interact Cardiovasc Thorac Surg. (2023) 36:ivad003. doi: 10.1093/icvts/ivad003, PMID: 36802255 PMC9931052

[26] Fang J Shi Y Du F Xue Z Cang J Miao C. The effect of Perineural dexamethasone on rebound pain after Ropivacaine single-injection nerve block: a randomized controlled trial. BMC Anesthesiol. (2021) 21:47. doi: 10.1186/s12871-021-01267-z, PMID: 33579199 PMC7879628

[27] TTH J JXC K Wing KJ Denomme J DI MI Huang SC. Development and internal validation of a multivariable risk prediction model for severe rebound pain after foot and ankle surgery involving single-shot popliteal sciatic nerve block. Br J Anaesth. (2022) 129:127–35. doi: 10.1016/j.bja.2022.03.030 35568510

[28] Woo JH Lee HJ Oh HW Lee JW Baik HJ Kim YJ. Perineural dexamethasone reduces rebound pain after Ropivacaine single injection Interscalene block for arthroscopic shoulder surgery: a randomized controlled trial. Reg Anesth Pain Med. (2021) 46:965–70. doi: 10.1136/rapm-2021-102795, PMID: 34535548

[29] Muñoz-Leyva F Cubillos J Chin KJ. Managing rebound pain after regional anesthesia. Korean J Anesthesiol. (2020) 73:372–83. doi: 10.4097/kja.20436, PMID: 32773724 PMC7533186

[30] Zahn PK Brennan TJ. Primary and secondary hyperalgesia in a rat model for human postoperative pain. Anesthesiology. (1999) 90:863–72. doi: 10.1097/00000542-199903000-00030 10078689

[31] Pogatzki-Zahn EM Segelcke D Schug SA. Postoperative pain-from mechanisms to treatment. Pain Rep. (2017) 2:e588. doi: 10.1097/pr9.0000000000000588, PMID: 29392204 PMC5770176

[32] Johnson ME. Neurotoxicity of lidocaine: implications for spinal anesthesia and neuroprotection. J Neurosurg Anesthesiol. (2004) 16:80–3. doi: 10.1097/00008506-200401000-00017, PMID: 14676575

[33] Perez-Castro R Patel S Garavito-Aguilar ZV Rosenberg A Recio-Pinto E Zhang J. Cytotoxicity of local anesthetics in human neuronal cells. Anesth Analg. (2009) 108:997–1007. doi: 10.1213/ane.0b013e31819385e1, PMID: 19224816

[34] Verlinde M Hollmann MW Stevens MF Hermanns H Werdehausen R Lirk P. Local anesthetic-induced neurotoxicity. Int J Mol Sci. (2016) 17:339. doi: 10.3390/ijms17030339, PMID: 26959012 PMC4813201

[35] Singh NP Makkar JK Chawla JK Sondekoppam RV Singh PM. Prophylactic dexamethasone for rebound pain after peripheral nerve block in adult surgical patients: systematic review, Meta-analysis, and trial sequential analysis of randomised controlled trials. Br J Anaesth. (2024) 132:1112–21. doi: 10.1016/j.bja.2023.09.022, PMID: 38501226

[36] Capdevila X Bringuier S Borgeat A. Infectious risk of continuous peripheral nerve blocks. Anesthesiology. (2009) 110:182–8. doi: 10.1097/ALN.0b013e318190bd5b, PMID: 19104185

[37] Hauritz RW Hannig KE Balocco AL Peeters G Hadzic A Børglum J. Peripheral nerve catheters: a critical review of the efficacy. Best Pract Res Clin Anaesthesiol. (2019) 33:325–39. doi: 10.1016/j.bpa.2019.07.015, PMID: 31785718

[38] Fu Z Zhang Y Zhou Y Li Z Wang K Li H. A comparison of paravertebral block, erector spinae plane block and the combination of erector spinae plane block and paravertebral block for post-operative analgesia after video-assisted thoracoscopic surgery: a randomised controlled trial. J Minim Access Surg. (2022) 18:241–7. doi: 10.4103/jmas.JMAS_277_20, PMID: 33885016 PMC8973475

[39] Alagoz A Findik G Sazak H Demiroz SM Baldemir R Ulger G. Non-intubated video-assisted Thoracoscopic surgery under combination of erector spinae plane block and thoracic paravertebral block. BMC Anesthesiol. (2022) 22:99. doi: 10.1186/s12871-022-01634-4, PMID: 35387585 PMC8985295

[40] Et T Basaran B Bilge A Yarımoğlu R Korkusuz M Tülüce İ. Rebound pain after Interscalene brachial plexus block for shoulder surgery: a randomized clinical trial of the effect of different multimodal analgesia regimens. Ann Saudi Med. (2023) 43:339–47. doi: 10.5144/0256-4947.2023.339, PMID: 38071444 PMC11182429

